# Effect of temperature, relative humidity, and time on the detection of swine RNA viruses (PRRSV, PEDV, IAV) inoculated onto filter papers

**DOI:** 10.3389/fcimb.2026.1753469

**Published:** 2026-05-08

**Authors:** Betsy Armenta-Leyva, Berenice Munguía-Ramírez, Yanqi Zhang, Jianqiang Zhang, Rolf Rauh, Luis G Giménez-Lirola, Jeffrey J Zimmerman

**Affiliations:** 1 Department of Veterinary Diagnostic and Production Animal Medicine, College of Veterinary Medicine, Iowa State University, Ames, IA, United States; 2 Department of Statistics, College of Liberal Arts and Sciences, Iowa State University, Ames, IA, United States; 3 Tetracore Inc., Rockville, MD, United States

**Keywords:** environmental surface, paper samplers, relative humidity, temperature, time, viral RNA stability

## Abstract

**Introduction:**

Filter papers have a long history of use in diagnostic specimen collection and storage, e.g., Guthrie cards, and recently, as leave-in-place environmental samplers in laboratory animal colonies. Paper offers a practical, low-cost solution to sample handling, but there is little data on the effect of temperature (T), relative humidity (RH), and time on target detection. The aim of this study was to evaluate the effect of T, RH, and time on the recovery of PRRSV, PEDV, and IAV RNA from 4 paper products under defined environmental conditions.

**Methods:**

In Study 1, 4 paper products were inoculated with each virus and subjected to one of 9 combinations of T (5 °C, 20 °C, 35 °C) × RH (< 20%, 40 - 65%, > 75%) for 0 to 7 days. In Study 2, paper products 3 and 4 were inoculated with PRRSV and PEDV and held at 20 °C × RH (< 20%, 40 - 65%, > 75%) for 0 to 28 days. The effect of environmental conditions on detectable viral RNA was determined by RT-qPCR with results normalized to efficiency-standardized Cqs (ECqs).

**Results and discussion:**

In Study 1, recovery of viral RNA differed among viruses and by temperature, but paper products performed similarly. In Study 2, both viruses were recovered from both paper products through the 28-day sampling period. However, higher RH (≥ 40% RH) was associated with increased loss of detectable RNA. At < 20% RH, no decay was observed for either virus on either paper. Overall, our findings indicate that multiple paper products are suitable for storage of viral specimens; albeit the optimal storage conditions would include low RH and low temperature.

## 1 Introduction

Effective environmental surveillance is increasingly recognized as critical to the prevention and control of swine viral diseases because many important swine pathogens are able to persist in the environment. For example, influenza A virus (IAV) has been shown to remain infectious in wetland water for up to 118 days under natural field conditions, and viral RNA detectable for 132 days (Reeves et al., 2020); foot-and-mouth disease virus has been recovered from dry hay after 20 weeks; and pseudorabies virus (PRV) can persist for up to 36 days on shelled corn and 18 days on steel surfaces (Pirtle and Beran, 1991). It follows that point-in-time environmental sampling has been used to surveil for a variety of swine pathogens, including African swine fever virus (Kwon et al., 2024), IAV (Garrido-Mantilla et al., 2019), porcine epidemic diarrhea virus (PEDV; Phillips et al., 2022), porcine circovirus type 2 (López-Lorenzo et al., 2019), porcine reproductive and respiratory syndrome virus (PRRSV; Melini et al., 2024), and *Salmonella* spp (Zewde et al., 2009). Typically, environmental surveillance involves collecting surface samples using commercial cleaning “wipes” or cotton swabs and testing for specific viral and bacterial targets, although complementary approaches such as bioaerosol sampling have also been explored within and around production facilities (Anderson et al., 2017).

The major weakness of this approach is that it cannot address the temporal and spatial heterogeneity of pathogens in the environment (Rehman et al., 2023; Seethapathy et al., 2008). Point-in-time sampling provides only a snapshot, which risks missing intermittent shedding events or uneven distribution of pathogens across surfaces. For example, O’Connor et al. (2006) quantified Salmonella in swine lairage pens and found concentrations varied both within and between pens, with some areas below detection and others exceeding 4 log CFU/ml. Alternatively, leave-in-place filter paper-based sampling addresses these dynamic heterogeneities by continuously accumulating target. Further, the practicality of this approach has been demonstrated in laboratory animal surveillance whereby samplers placed in cages have replaced sentinel mice for detecting relevant pathogens (O’Connell et al., 2021; Körner et al., 2019; Luchins et al., 2020; Lupini et al., 2023; Mailhiot et al., 2020; Miller et al., 2016; Niimi et al., 2018; Simmons et al., 2024; Williams et al., 2024; Winn et al., 2022).

In its own right, filter paper has a long history of use in human and veterinary medicine for sample collection, transport, and storage, particularly in settings with limited cold-chain infrastructure (Guthrie and Susi, 1963; Smit et al., 2014). Since the early 1960’s, dried blood spots (DBS) have been collected from newborn infants on cellulose-based “Guthrie cards” for phenylketonuria screening (Guthrie and Susi, 1963). Over time, the use of filter papers has broadened to include the collection of environmental samples for nucleic acid detection using a variety of filter paper types (Cha et al., 2023; Corchis-Scott et al., 2021; Dubelko et al., 2018; Gerwin et al., 2017; Hanson et al., 2021; Williams et al., 2024). Although filter paper sampling is increasingly used in diagnostics and surveillance due to its low cost, ease of handling, and efficacy, most studies on the use of filter paper have largely focused on temperature and time effects. For example, Guzman et al. (2005) reported the detection of Venezuelan equine encephalitis RNA for up to 90 days using filter papers spotted with mouse brain homogenate and held at ~25 °C. Prado et al. (2005) reported the detection of dengue virus RNA for 9 weeks from filter paper inoculated with spiked human blood and held at -70 °C, 4 °C, or ~25 °C. Specifically, for swine viruses, Linhares et al. (2012) demonstrated that PRRSV RNA could be reliably detected by RT-PCR from serum and tissue samples stored on FTA cards and held at 4 °C or 25 °C for up to 14 days. Similarly, Braae et al. (2015) detected African swine fever virus (ASFV) DNA from blood samples stored on FTA cards for 4 to 6 months at ambient temperatures (15–30 °C).

Currently, the detection of target pathogens over time as a function of the type of filter paper and the environmental conditions is poorly characterized. Therefore, the goal of this study was to compare four filter paper products in terms of the effect of temperature, relative humidity (RH), and time on the detection of PRRSV, PEDV, and IAV using reverse transcriptase real-time PCR (RT-qPCR).

## 2 Materials and methods

### 2.1 Experimental design

A factorial experimental design was used to systematically evaluate the independent and interactive effects of temperature, relative humidity, and time on viral detection across multiple paper types and viruses under conditions representative of swine production environments.

In Study 1 (temperature × RH; evaluated across multiple paper types, viruses, and time points), 4 paper products (1) Whatman^®^ filter paper grade 903, (2) FTA^®^ Cards, (3) Polyester filter paper (Optimice Cage Reemay Filter Media), and (4) Swiffer^®^ dry cloth) were inoculated with PRRSV, PEDV, and IAV, and subjected to one of 45 experimental conditions accounting for all combinations of 3 levels of temperature (5, 20, 35 °C), 3 ranges of RH (< 20%, 40 - 65%, > 75%) and 5 collection time points (0 or untreated, 1, 3, 5, and 7 d).

In Study 2 (RH × time; conducted at a constant temperature over an extended time course), paper products 3 and 4 were inoculated with PRRSV and PEDV, held at 20 °C, subjected to one of 3 RH conditions (< 20%, 40 - 65%, > 75%), and sampled at 0 (untreated), 3, 7, 10, 14, 21, and 28 days post inoculation (DPI). For both studies, viral targets were eluted from paper swatches using PCR-grade water for cellulose-based papers and lysis buffer for polyester-based papers. Eluates were then tested by RT-qPCR.

### 2.2 Paper products

1. Whatman^®^ filter paper (Grade 903) – Contains a high percentage of alpha cellulose (98%). Commonly used for biological sample collection and analysis (catalog number: WHA10534612; Millipore Sigma, Burlington, MA, USA).
2. QIAcard™ FTA™ Classic cards – Cotton-based cellulose paper treated with proprietary chemicals to lyse cells, denature proteins, and stabilize nucleic acids (catalog no. WHAWB120205; Millipore Sigma).
3. Reemay^®^ polyester filter media (Grade 2024) – Spunbond polyester matrix characterized by high strength and stability (catalog no. C79070FLT; Animal Care Systems; Centennial, CO, USA).
4. Swiffer^®^ dry cloth – A nonwoven blend of polyester and polypropylene. Primarily intended for cleaning applications (catalog no. S-16029; Uline, Pleasant Prairie, WI, USA).

### 2.3 Viruses

Three viruses (PRRSV, PEDV, IAV) were included overall. PRRSV isolate MN-184 (GenBank accession no. EF488739) was propagated in African green monkey kidney (MARC-145) cells to a concentration of 6 × 10^4^ median tissue culture infectious dose (TCID_50_) per ml using a protocol described elsewhere (Neat et al., 2021). PEDV isolate USA/NC35140/2013 (GenBank accession no. KM975735) was propagated in Vero cells (ATCC CCL-81) to a concentration of 1.7 × 10^5^ TCID_50_ per ml, as described elsewhere (Chen et al., 2014). This PEDV isolate was originally derived from piglet feces submitted to the Iowa State University Veterinary Diagnostic Laboratory (ISU VDL) for routine diagnostic testing (Chen et al., 2016). Influenza A/swine/Iowa/A01778877/2016 (IA/16, 1A.3.3.3 gamma H1N1) was propagated in Madin-Darby canine kidney (MDCK) cells to a concentration of 5 × 10^7^ TCID_50_ per ml, as described by Zhang and Gauger (2020).

### 2.4 Treatments

To conduct the studies, PRRSV, PEDV, and IAV stock solutions were diluted 1:16, 1:4, and 1:4096, respectively, using swine oral fluids to achieve a Cq value of ~20. The oral fluid diluent was collected from 12- to 16-week old pigs held under biosafety level 3 research conditions. As described by Cheng et al. (2020), serum samples from these pigs were antibody negative for PRRSV, pseudorabies virus, and IAV and oral fluid samples were RT-qPCR negative for PRRSV, PEDV, and IAV. Thereafter, viral inoculums (300 μl) were deposited at different spots on individual paper swatches (5.1 cm × 3.8 cm; 2” × 1.5”). For all experimental conditions, three replicate swatches were prepared. Individual inoculated swatches were dried (5 h) in a 25 °C incubator (NAPCO 6301; Precision Scientific, Chicago, Illinois), placed inside tissue cassettes (CellPath^®^ Super Mega Hex Slim Cassette, Ted Pella, Inc, Redding, CA, USA), and held under defined conditions of temperature (5 °C, 20 °C, 35 °C) × RH (< 20%, 40 to 65%, > 75%). The temperature ranges were selected to represent cold, moderate, and warm conditions typical of swine productions and sample storage conditions.

An environmental chamber (Percival Advanced Intellus Environmental Controller 130NLX, Percival Scientific, Inc, Perry, IA) was used to maintain specific temperatures (5 °C, 20 °C, 35 °C). The chamber was maintained at 75 - 80% RH using a targeted-vapor-delivery humidifier (CloudForge™ T7, AC Infinity Inc., City of Industry, CA, USA) attached to the chamber. The internal RH of the chamber was monitored with a corded sensor probe and programming controller (Controller 69 PRO+, AC Infinity Inc.) set to activate the vaporizer if the internal RH was ≤ 70%.

At each temperature, 3 distinct RH microenvironments were established within the environmental chamber using 3 airtight containers (15 l, Hanamya, Hauppauge, NY, USA). A low RH (< 20%) microenvironment was maintained by inclusion of ~80 gr of zeolite desiccant (4A Molecular Sieve, Wisesorb^®^, Marlton, NJ, USA) in one of the 3 containers. Mid-RH (40 to 65%) was sustained by placing ~20 gr of calcium montmorillonite desiccant (Wisesorb^®^) in a second container. High RH (> 75%) microenvironment was created by placing a plastic beaker containing a saturated sodium chloride solution (Greenspan, 1976) (50–60 gr NaCl (Millipore Sigma) and 25 ml of PCR-grade water) in the third container. Continuous readings from hygrometers placed inside each container (Fisherbrand™ Memory-Loc™ Datalogging Traceable™ Hygrometers, Thermo Fisher Scientific, Inc.) were used to monitor RH and adjust desiccants, if required.

After inoculation and drying, swatches were placed within microenvironments, collected at designated time points, placed in vacuum-sealed bags (VacMaster^®^ Vacuum chamber pouches, and VacMaster^®^ VP215C, Ary, Inc., Kansas City, MO), and stored at -80 °C until tested for viral RNA. To provide a baseline of viral RNA detection over time, positive controls, i.e., individual aliquots (300 μl) of PRRSV (1:16), PEDV (1:4), or IAV (1:4096) in 2.5 ml cryovials (Simport™ Scientific Cryovial™, Thermo Fisher Scientific, Inc., Waltham, Massachusetts), were subjected to the same temperature treatments and collection time points as the inoculate test swatches and stored at -80 °C until tested for RNA.

**Figure 1:**
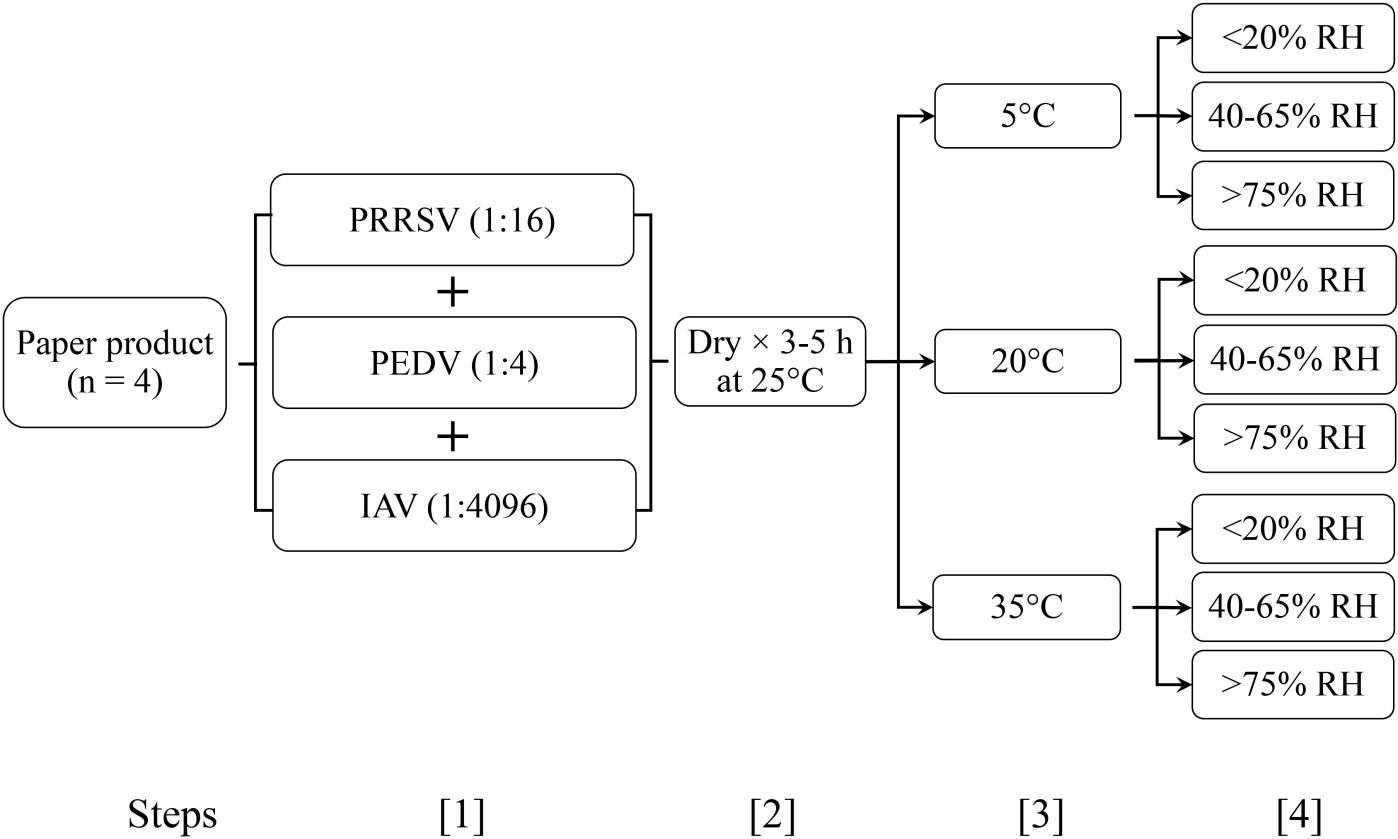
Study 1 (temperature × RH). 1. Swatches (paper products 1, 2, 3, 4) were inoculated with 300 μl of each virus; 2. Swatches were dried (25 °C incubator, 5 h); 3 - 4. Swatches were held under a defined temperature and relative humidity (RH); 5. Swatches were removed at 1, 3, 5, and 7 days and stored at -80 °C. Each combination of paper product, temperature, and RH was tested for RNA in triplicate.

In Study 1 (temperature × RH), 4 paper products were evaluated across all combinations of temperature × RH. In addition to the day 0 sampling, test swatches and positive controls were collected at 4 time points, i.e., 1, 3, 5, and 7 days post-inoculation. For each of the 4 paper products, a total of 111 test swatches were prepared, i.e., 3 temperature levels × 3 RH ranges × 4 sampling points × 3 replicates plus an untreated control (**Figure 1**).

**Figure 2:**
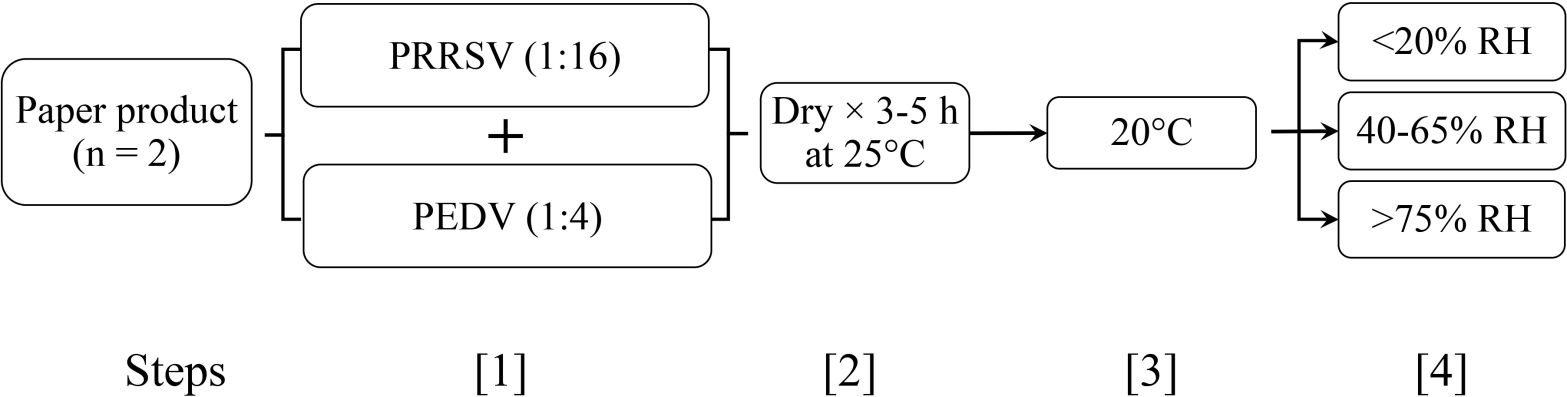
Study 2 (RH × time). 1. Swatches (paper products 3 and 4) were inoculated with 300 μl of each virus; 2. Swatches were dried (25 °C incubator, 5 h); 3 - 4. Swatches were held at 20 °C and a defined relative humidity (RH); 5. Swatches were removed at 3, 7, 10, 14, 21, 28 days and stored at -80 °C. Each combination of paper product, temperature, and RH was tested in triplicate.

In Study 2 (RH × time), paper products 3 and 4 were evaluated at 20 °C under all 3 RH conditions. In addition to the day 0 sampling, test swatches and positive controls were collected at 6 time points: 3, 7, 10, 14, 21, and 28 days post-inoculation. For each of the 2 paper products, a total of 57 test swatches were prepared, i.e., 1 temperature level × 3 RH ranges × 6 sampling points × 3 replicates plus an untreated control (**Figure 2**).

### 2.5 Elution of viral RNA from paper products

In Study 1, paper swatches were removed from -80 °C storage and held at 4 °C overnight (Weiser et al., 2018). To recover viral RNA, swatches were placed in 50 ml conical tubes (Corning Life Sciences) containing elution buffer (2 ml). The tube was manually inverted (3–5 times) to ensure that the paper was saturated with the elution buffer and then incubated at 4 °C for 1 hour. Based on previously reported data (Armenta-Leyva et al., 2025), PCR-grade water was used to elute viral RNA from paper products 1 and 2 and general lysis buffer (Buffer G2, Qiagen, Hilden, Germany) for paper products 3 and 4. In study 2, paper swatches were placed in plastic filter bags (Whirl-Pak^®^ Homogenizer Blender Filter Bag, Millipore Sigma) with general lysis buffer (2 ml), agitated with a stomacher blender (Seward^®^ Stomacher 80 Biomaster Lab Blender, Cole-Parmer, Vernon Hills, IL), incubated at 4 °C for 1 hour, and then reagitated. To match the testing conditions of test swatches, positive controls (300 μl) were thawed at 4 °C (overnight) and added directly to 2 ml of either PCR-grade water or lysis buffer. Eluates from test swatches and controls were stored at -80 °C until assayed by RT-qPCR.

### 2.6 RT-qPCR reference standards

To normalize PCR results, in-house reference standards were run on every RT-qPCR plate. PRRSV reference standards were prepared by reconstituting a lyophilized 10-dose PRRSV modified live virus (MLV) vaccine (Ingelvac^®^ PRRSV MLV, Boehringer Ingelheim Vetmedica, Inc., Duluth, GA, USA) in 20 ml of PRRSV-free oral fluids. IAV reference standards were generated from a commercial 50-dose IAV killed virus vaccine (FluSure XP^®^, Zoetis) reconstituted with 100 ml of IAV-free oral fluids. Each solution was serial ten-fold diluted and the dilution producing a quantification cycle (Cq) value of ~30 was selected for use (1:1,000 for PRRSV, 1:10,000 for IAV). PEDV reference standards were prepared from a commercial PEDV inactivated virus vaccine (Zoetis, Florham Park, NJ, USA). The vaccine was thoroughly mixed and then 15 ml were centrifuged (3,300 × *g* for 30 min) in a conical tube (Corning Life Sciences). The supernatant was ten-fold serially diluted and the dilution (1:10) that produced a Cq of ~30 was selected for use.

### 2.7 RNA extraction and RT-qPCR

Nucleic acids were extracted from eluates using MagMAX™ Pathogen RNA/DNA Kit (Thermo Fisher Scientific, Inc.) on the KingFisher™ Flex automated platform using a protocol optimized for low-cell content specimens. Briefly, a lysis/binding mixture comprising 125 μl of lysis/binding concentrate, 2 μl of carrier RNA, 125 μl of 100% isopropanol, and 6 μl of VetAlert™ IC RNA Control (Tetracore Inc., Rockville, MD), was combined with 100 μl of sample eluate and 20 μl of magnetic bead suspension. Nucleic acids were eluted in 90 μl of MagMAX™ elution buffer. Every nucleic acid extraction plate included 82 research samples, 4 PRRSV reference standards, 4 IAV reference standards, 4 PEDV reference standards, and 2 extraction controls (1 positive, 1 negative).

Immediately thereafter, sample extracts were tested for nucleic acids. PRRSV RNA detection was done using the EZ-PRRSV™ MPX 4.0 Master Mix and Enzyme (Tetracore, Inc.), IAV RNA detection was performed with the EZ-Universal Flu A 2.0 RT-PCR (Tetracore, Inc.), and PEDV RNA with the EZ-PED/TGE/PDCoV MPX 1.1™ (Tetracore, Inc.). Each RT-qPCR mixture consisted of 17.25 μl of EZ-PRRSV™ MPX 4.0, EZ-Universal Flu A 2.0, or PED/TGE/PDCoV MPX 1.1™ reagents, and 0.75 μl of enzyme blend. To this mixture was added 7 μl of sample extract, negative amplification control (NAC; PCR-grade water) or positive amplification control (PAC) for a final reaction volume of 25 μl. PAC were specific for each pathogen: EZ-PRRSV™ MPX 4.0 Control Set (Tetracore, Inc.) for PRRSV, EZ-Universal Flu A 2.0 Control Set (Tetracore, Inc.), or EZ-PED/TGE/PDCoV MPX 1.1 Control Set (Tetracore, Inc.).

Each plate included 1 PAC and 1 NAC, extracts from the 2 extraction controls, and extracts from the target-specific reference standards. Specifically, 4 PRRSV reference standard extracts were included on each PRRSV RT-qPCR plate, 4 IAV reference standard extracts on IAV RT-qPCR plates, and 4 PEDV reference standard extracts on PEDV RT-qPCR plates. Reactions were run on the Applied Biosystems^®^ 7500 Fast thermal cycler (Thermo Fisher Scientific, Inc.) under the following conditions: 48 °C for 15 m, 95 °C for 2 m, and 45 cycles of 95 °C for 5 s and 60 °C for 40 s. Results were evaluated and reported as raw Cqs with the Design and Analysis Software (DA2 Software v2.8.0; Thermo Fisher Scientific, Inc.).

### 2.8 Normalization of RT-qPCR results

To account for variation in sampling and testing, Cq results were normalized prior to data analysis by converting them to “efficiency standardized Cqs (ECqs)” using Equation 1.

(1) $$\text{Efficiency standardized Cq}(\text{ECq})={\text{E}}^{-}\Delta \text{Cq}={\text{E}}^{-(\text{Sample Cq}-\text{Mean Cq of reference standards})}$$

In Equation 1, E represents the PCR amplification efficiency and ΔCq is the difference between a sample’s Cq and the mean Cq of the reference standards included on each plate (Armenta-Leyva et al., 2024; Pfaffl, 2001). Amplification efficiency can be expressed either as a ratio (number of target amplicons at the end of a PCR cycle divided by the number at the beginning) or as a percentage. An efficiency of 2 (or 100%) would represent doubling at each cycle, i.e., perfect amplification. To normalize the results, E values (expressed as ratios) for each reference standard were estimated from the raw fluorescence data using web-based software LinRegPCR (https://www.gear-genomics.com/rdml-tools; Untergasser et al., 2021). E in Equation 1 was replaced with the arithmetic mean of the 4 reference standards’ estimated E values, with any values exceeding 2 truncated at 2. For samples with undetermined Cq values, i.e., a sample with no amplification, the final Cq value was defaulted to 45.

ECqs are a measure of the fold change in target concentration between a sample and the reference standard. A sample with a Cq of 28, ran on a plate with a reference standard mean efficiency of 92% (E = 1.92) and a mean Cq of 30, would have an ECq of 3.69 (ECq = E^-ΔCq^ = 1.92^–(28–30)^ = 3.69). This value means the target concentration in the sample is 3.69 times that of the reference standard. In our study, ECqs were transformed to the cube root to help condense the range of values. Thus, the reported ECq values reflect the cube root of the calculated fold change. For instance, an ECq of 3 corresponds to a fold change of 3^3^ = 27, meaning the sample contains 27 times the amount of target present in the reference standard. Based on previous work evaluating PRRSV detection in swine oral fluids (Armenta-Leyva et al., 2026), an RT-qPCR cutoff of ECq ≥ 0.1 was used to classify samples as positive.

### 2.9 Analysis

In Study 1 (temperature × RH), ECq responses for each pathogen were modeled in RStudio v4.2.2 (RStudio Team, 2020) as a function of paper product, temperature, RH, and time. The initial full linear regression model included all main effects and interactions. Non-significant variables and interactions were removed during model simplification; however, key experimental factors of primary interest, i.e., paper product, were retained in the final model regardless of statistical significant. The final regression model for each pathogen included paper product, temperature, time, the positive control as the reference level, and RT-qPCR plate as a random effect. For each pathogen, type III ANOVA was used to assess the significance of each predictor.

In Study 2 (RH × time), each virus was analyzed using a nonlinear exponential decay model (Equation 2):

(2) $$y=\text{A}\times {\text{e}}^{\left(-\text{k}\times \text{t}\right)}+\text{C}$$

where A is the initial signal amplitude, k is the decay rate constant, and C is the residual baseline. Models were fitted independently in RStudio v4.2.2 (RStudio Team, 2020) with package minpack.lm (Elzhov et al., 2022) using nonlinear least squares for each combination of paper product and RH. To compare decay rates (k) between paper products under the same RH, Wald-type z-tests were conducted using estimated k values and their standard errors, assuming independent model fits.

## 3 Results

**Figure 3:**
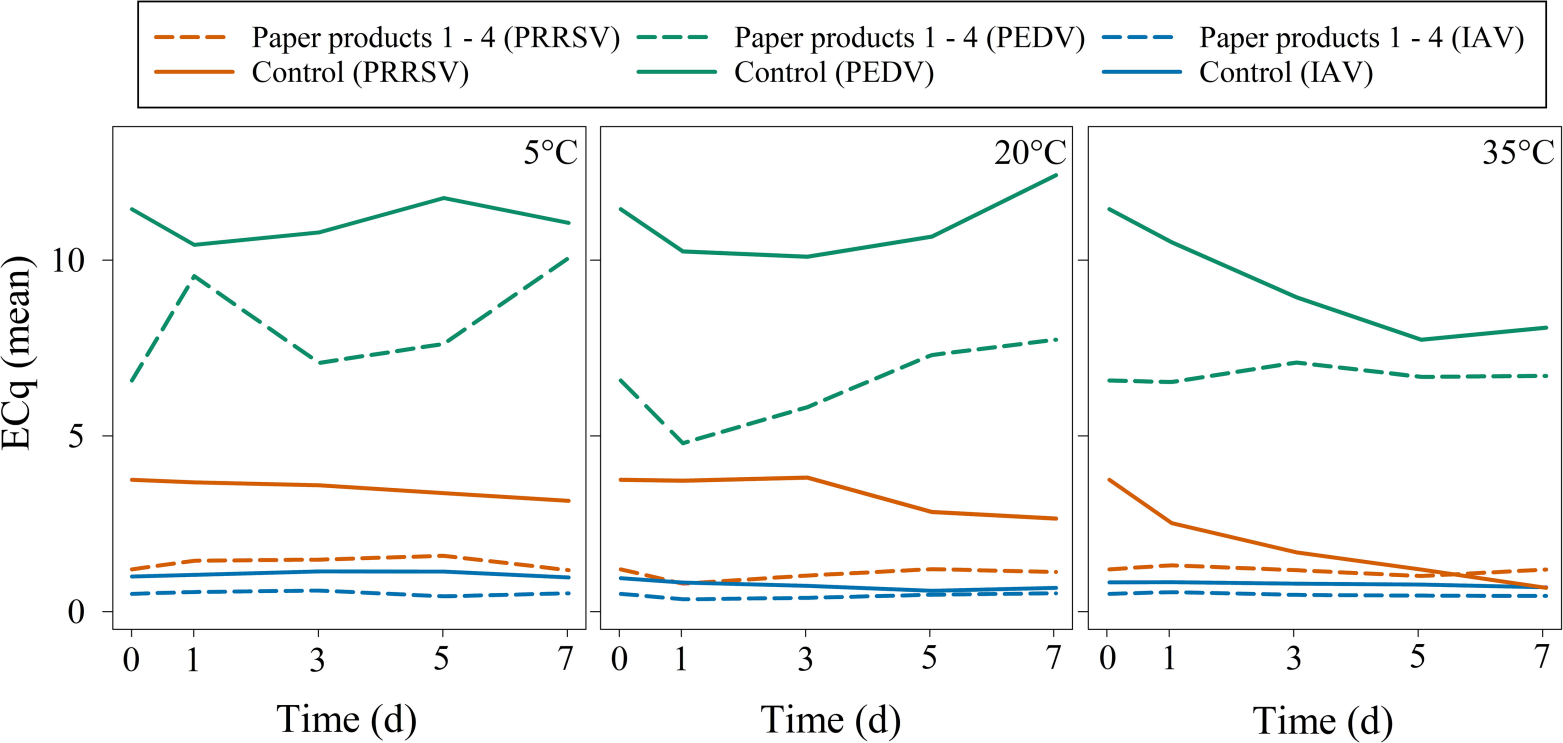
Study 1 (temperature × RH) – Mean ECq responses for PRRSV, PEDV, and IAV over a 7-day sampling period aggregated across relative humidity and paper products at each temperature. ECq means were calculated from positive results using an RT-qPCR cutoff of ECq ≥ 0.1. Within pathogen, time was not a significant predictor of ECq values (p > 0.05, linear-mixed effects model).

In Study 1 (temperature × RH), linear regression modeling using the control as the baseline showed that temperature significantly influenced ECq responses for all three viruses (p < 0.05). In contrast, RH was removed from the models due to lack of significance. Time did not significantly affect ECq values for any virus (p > 0.05) over the 7-day sampling period. Accordingly, ECq values were aggregated across paper products to emphasize the absence of temporal change (**Figure 3**).

**Figure 4:**
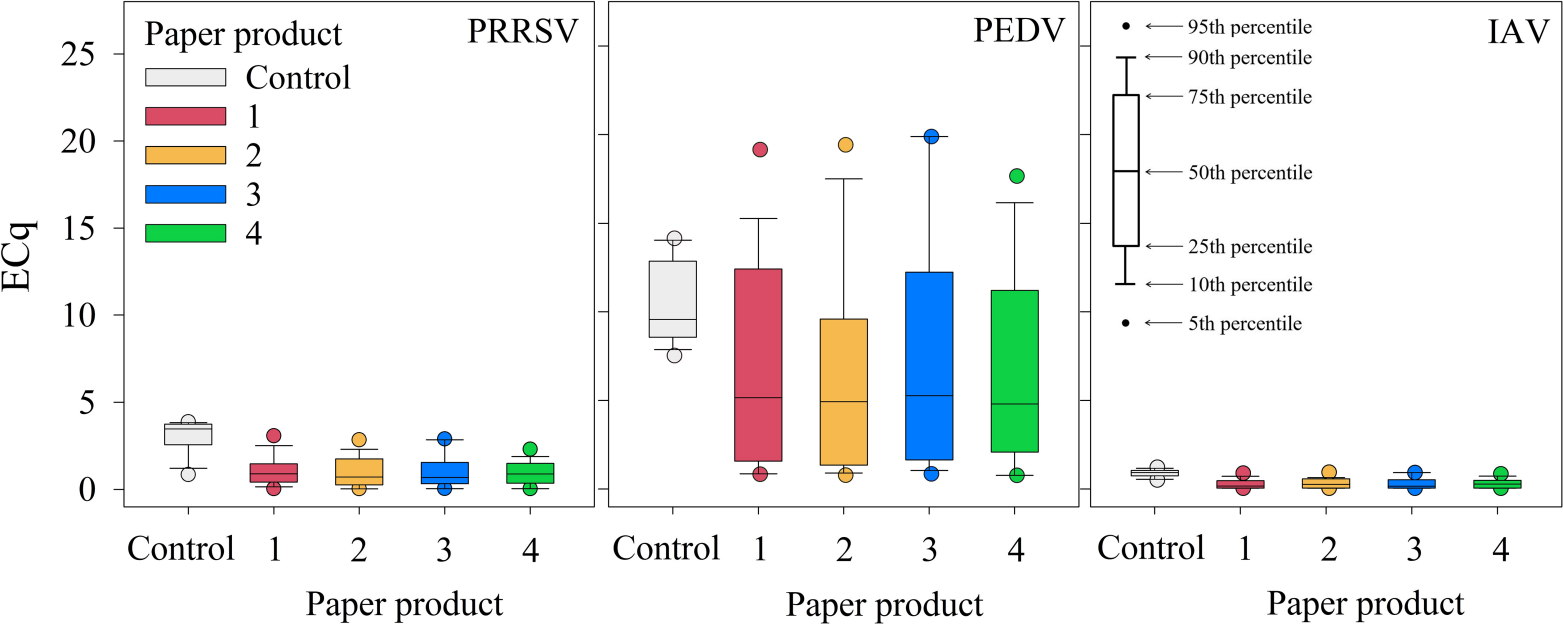
Study 1 (temperature × RH) – ECq boxplot distributions for PRRSV, PEDV, and IAV for paper products and the positive controls aggregated across environmental conditions.

**Table 1** summarizes ECq responses by paper product, temperature, and virus across all time points and RH conditions. Controls were 100% positive across temperatures and viruses. For PRRSV and IAV but not PEDV, the positive control mean ECq value was significantly higher than all paper products at each temperature (p < 0.05) (**Table 2**). Detection rates varied by paper products ranging from 80.0% to 97.8% for PRRSV, 97.8% to 100% for PEDV, and 46.7% to 80.0% for IAV; however, no significant differences were detected between paper products for any virus (p > 0.05). Across paper products, the median ECq values were lower than those of the positive control for all viruses: 0.682 to 0.891 for PRRSV (control: 3.46), 4.80 to 5.27 for PEDV (control: 9.57), and 0.157 to 0.277 for IAV (control: 0.923) (**Figure 4**).

**Table 1:** Study 1 (temperature × RH) – proportion of PRRSV, PEDV, and IAV positive paper products by environmental temperature^1^.

Temperature	Paper product	PRRSV	PEDV	IAV
5 °C	Control	100% 3.51 (0.04)^2^	100% 11.1 (0.42)	100% 1.06 (0.02)^2^
	Paper product 1	91.1% 1.24 (0.18)	100% 7.41 (0.87)	64.4% 0.46 (0.05)
	Paper product 2	84.4% 1.65 (0.15)	100% 10.1 (1.10)	80.0% 0.55 (0.05)
	Paper product 3	88.9% 1.29 (0.27)	100% 7.56 (0.10)	51.1% 0.51 (0.06)
	Paper product 4	86.7% 1.32 (0.19)	100% 7.62 (0.92)	68.9% 0.55 (0.06)
20 °C	Control	100% 3.42 (0.11)^2^	100% 11.0 (0.58)	100% 0.78 (0.04)^2^
	Paper product 1	88.9% 1.04 (0.12)	100% 6.10 (0.84)	60.0% 0.39 (0.05)
	Paper product 2	86.7% 1.00 (0.12)	97.8% 5.49 (0.82)	53.3% 0.48 (0.05)
	Paper product 3	86.7% 1.28 (0.17)	100% 8.43 (1.01)	66.7% 0.51 (0.06)
	Paper product 4	86.7% 0.96 (0.10)	100% 5.76 (0.71)	55.6% 0.45 (0.05)
35 °C	Control	100% 2.03 (0.22)^2^	100% 9.43 (0.44)	100% 0.78 (0.04)^2^
	Paper product 1	97.8% 1.27 (0.17)	100% 7.43 (0.95)	62.2% 0.49 (0.06)
	Paper product 2	80.0% 1.00 (0.12)	100% 5.54 (0.76)	53.3% 0.45 (0.05)
	Paper product 3	86.7% 1.33 (0.16)	100% 6.70 (1.01)	46.7% 0.62 (0.08)
	Paper product 4	88.9% 1.09 (0.10)	100% 7.20 (0.82)	75.6% 0.42 (0.04)

^1^ Results reported as proportion positive, mean ECq of positives, and standard error of the mean. Proportions based on a denominator of 45 samples for each paper product and a denominator of 30 for control samples.

^2^ Pairwise comparisons showed that positive control mean ECq results differed from paper products (p < 0.05, linear mixed-effects model). No significant differences were detected in the proportions of PRRSV, PEDV, and IAV RNA-positive paper products within environmental temperature.

**Table 2:** Study 1 (temperature × RH) – Type III analysis of variance.

Virus	Source of variation	Sum of squares	Mean square	Degrees of freedom	F-value	P-value
PRRSV	Paper	273.193	68.298	4	615	<0.05
	Time	1.229	1.229	1	615	0.276
	Temperature	12.589	6.295	2	615	0.002
PEDV	Paper	96.224	24.056	4	0.809	0.534
	Time	213.579	106.7789	2	3.591	0.295
	Temperature	32.568	32.568	1	1.095	<0.05
IAV	Paper	3.455	0.8863	4	10.370	<0.05
	Time	1.112	0.556	2	6.675	0.771
	Temperature	0.007	0.007	1	0.084	<0.05

Derived from a linear regression model for each pathogen that included paper product, temperature, time, and the positive control as the reference level.

In Study 2 (RH × time), 112 of 114 swatches (98.2%) collected from DPI 0 to 28 were positive for PRRSV RNA. The two negative swatches were collected on DPI 28; one held at 40 - 65% RH (paper product 3) and one held at > 75% RH (paper product 4). For PEDV, 114 of 114 swatches (100%) collected from DPI 0 to 28 were RNA positive.

**Figure 5:**
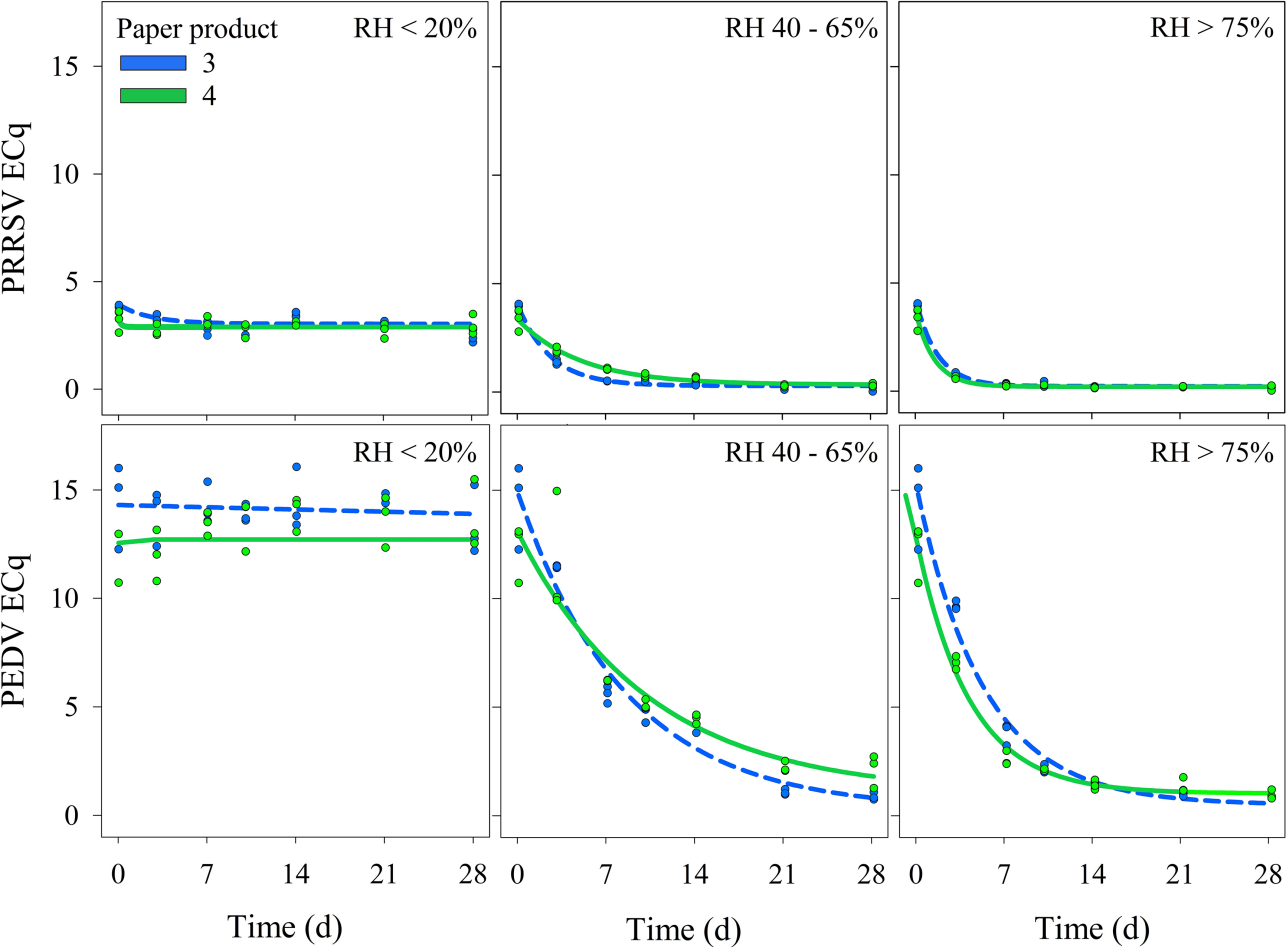
Study 2 (RH × time) – Comparison of the effect of relative humidity (RH) and time on the detection of PRRSV and PEDV RNA from two paper products. Each panel shows observed ECq values (circles) and predicted values (lines) based on a nonlinear exponential decay model. ECq trend lines are shown for conditions where the analyses found no evidence of decay.

Nonlinear exponential decay models derived for each combination of (virus × paper product × RH) are listed in **Table 3** and the predicted responses are shown in **Figure 5**. Two combinations showed no evidence of decay, i.e., paper product 4 inoculated with PRRSV or PEDV and held at <20% RH. Based on Wald-type z-tests, no statistically significant differences were observed between paper products under matched RH conditions, except for PRRSV at 40–65% RH which showed a significantly greater decay rate for paper product 3 compared to product 4 (p < 0.05). In general, decay rates increased with RH for both viruses. This pattern is reflected in both the fitted equations in **Table 3** and the decay trajectories shown in **Figure 5**, where increasing RH corresponds to steeper curves and larger decay rates (k).

**Table 3:** Study 2 (RH × time) – fitted exponential decay equations for PRRSV and PEDV by paper product and relative humidity (RH).

		Nonlinear exponential decay equation		
Virus	RH	Paper product 3	Paper product 4	Δ*k*
PRRSV	< 20%	$\text{y}=0.89\times {\text{e}}^{\left(-0.390\times \text{t}\right)}+3.040$	No evidence of decay.	--
	40 - 65%	$\text{y}=3.62\times {\text{e}}^{\left(-0.389\times \text{t}\right)}+0.290$	$\text{y}=2.96\times {\text{e}}^{\left(-0.200\times \text{t}\right)}+0.341$	0.19^1^
	> 75%	$\text{y}=3.69\times {\text{e}}^{\left(-0.579\times \text{t}\right)}+0.227$	$\text{y}=3.12\times {\text{e}}^{\left(-0.675\times \text{t}\right)}+0.211$	0.10
PEDV	< 20%	$\text{y}=14.3\times {\text{e}}^{\left(-0.001\times \text{t}\right)}+0.002$	No evidence of decay.	--
	40 - 65%	$\text{y}=14.5\times {\text{e}}^{\left(-0.117\times \text{t}\right)}+0.266$	$\text{y}=12.0\times {\text{e}}^{\left(-0.096\times \text{t}\right)}+1.010$	0.02
	> 75%	$\text{y}=14.3\times {\text{e}}^{\left(-0.187\times \text{t}\right)}+0.493$	$\text{y}=11.4\times {\text{e}}^{\left(-0.238\times \text{t}\right)}+1.020$	0.05

^1^ Decay was significantly greater compared to paper product 4 (p < 0.05, Wald-type z-test).

## 4 Discussion

Filter paper has been demonstrated to stabilize a variety of diagnostic targets under diverse storage conditions. Antibodies and viral nucleic acids have been shown to remain detectable on cellulose-based papers under a range of temperatures and durations. For example, antibodies from coyote blood collected on Nobuto strips were detected for > 2 years when stored at −20 °C or 4 °C with desiccant (Bevins et al., 2016). Similarly, ASFV-positive whole blood samples spotted onto filter paper (Whatman^®^ 903) remained PCR-positive after 9 months at 22 - 25 °C or 37 °C (Michaud et al., 2007), and SARS-CoV-2 RNA on FTA cards remained stable for at least three weeks between -20 °C and 37 °C (Kriegshäuser et al., 2021). A systematic review further supports the use of FTA cards for viral RNA preservation across diverse pathogens (Cardona-Ospina et al., 2019). Collectively, these findings indicate that filter paper can be a reliable storage matrix for diagnostic specimens, depending on the analyte (protein, antibody, nucleic acid) and storage conditions, i.e., temperature, humidity, and storage medium.

The aim of the present study was to evaluate the effect of temperature, relative humidity, and time on the recovery of PRRSV, PEDV, and IAV RNA from four virus-inoculated filter paper products. A number of studies have reported the effect of temperature × time on PRRSV, PEDV, and IAV infectivity or detectability, albeit the experimental designs, data analysis, and response units differed among studies (**Table 4**). Inactivation by temperature × time varied markedly by virus and matrix, but generally speaking, IAV was the most labile of the three, PRRSV showed intermediate resistance, and PEDV the most resistant. Typically, colder conditions supported longer stability, while increasing temperatures accelerated virus inactivation. These findings aligned with broader principles of RNA virus biology in the sense that polymerase activity, RNA secondary structure stability, and lipid envelope phase transitions are all affected by temperature. By way of illustration, exposure of SARS-CoV-2 viral particles to 34 °C induced structural degradation, while at 22 °C they remained intact (Bisht and te Velthuis, 2022).

**Table 4:** Effect of temperature × time on IAV, PRRSV, or PEDV infectivity or RNA detection on various surfaces.

Pathogen	Temperature	Environment	Findings	Citation
IAV	4 °C	Distilled water	Infectious after 170 days	Poulson et al., 2016
		PET^1^, stainless steel, glass	RNA detected up to 24 hours	Meng et al., 2022
	25 °C	Wastewater	RNA detected up to 6 days	Qiu et al., 2024
		Plastics^1^	RNA detection declined to 10% after 14 days	Meng et al., 2022
	37 °C	Distilled water	Infectious up to 3 days	Poulson et al., 2016
		Plastics^1^	Infectivity lost after 24 hours	Meng et al., 2022
PRRSV	~4 °C	Concrete, cardboard	RNA detection up to 12 hours	Jacobs et al., 2010
		Polystyrene foam, metal	RNA detection up to 4 hours	Lugo Mesa et al., 2024
		Serum	RNA detection up to 168 hours	Munguía-Ramírez et al., 2023
		Oral fluid	RNA decay of -0.03 ECq/24 hours	Munguía-Ramírez et al., 2023
		Feces	RNA decay of -0.05 ECq/24 hours	Munguía-Ramírez et al., 2023
	10-20 °C	Metal, rubber, cardboard	RNA detection up to 1–8 hours	Jacobs et al., 2010
	20 °C	Serum	RNA decay of -0.02 ECq/24 hours	Munguía-Ramírez et al., 2023
		Oral fluid	RNA decay of -0.05 ECq/24 hours	Munguía-Ramírez et al., 2023
		Feces	RNA decay of -0.09 ECq/24 hours	Munguía-Ramírez et al., 2023
	30 °C	Serum	RNA decay of -0.11 ECq/24 hours	Munguía-Ramírez et al., 2023
		Oral fluid	RNA decay of -0.08 ECq/24 hours	Munguía-Ramírez et al., 2023
		Feces	RNA decay of -0.09 ECq/24 hours	Munguía-Ramírez et al., 2023
		Cardboard	Infectious virus undetectable after 6 hours	Mil-Homens et al., 2024
	50 °C	Aluminum	Infectious virus undetectable after 15 minutes	Mil-Homens et al., 2024
PEDV	4 °C	Polystyrene foam, cloth	Infectious virus recovered up to 15 days	Kim et al., 2018
		Plasma	Infectious virus recovered at 7 days	Pujols and Segalés, 2014
	4-8 °C	Seawater	RNA detection up to 16 days	Contrant et al., 2023
	20 °C	Cardboard, aluminum	Infectious virus recovered up to 36 hours	Mil-Homens et al., 2024
	22 °C	Plasma	No infectious virus was recovered after 7 days	Pujols and Segalés, 2014
	25 °C	Polystyrene foam, cloth,	No infectious virus was recovered after 2 days	Kim et al., 2018
	30 - 50 °C	Cardboard	No infectious virus after 12 hours	Mil-Homens et al., 2024
	30 - 40 °C	Aluminum	No infectious virus after 6 hours	Mil-Homens et al., 2024
	50 °C	Aluminum	Infectious virus recovered up to 15 minutes	Mil-Homens et al., 2024

^1^ Plastics included polypropylene, polyethylene, polystyrene, polyethylene terephthalate (PET), polyvinyl chloride, polymethyl methacrylate.

The effect of RH on virus stability is often excluded from temperature × time studies - despite the fact that RH can markedly affect viral stability. In aerosols, IAV half-lives in air varied with humidity, i.e., ~6 days at <30% RH, ~13 days at 30 - 70% RH, and ~9 days at >70% RH (Irwin et al., 2011). Hermann et al. (2007) found that the stability (half-life) of aerosolized PRRSV increased as relative humidity declined, e.g., at 10 °C and 10% RH half-life was estimated at 136 hours vs 74 hours at 10 °C and 90% RH. The effect of RH on PEDV has not been characterized, but studies on transmissible gastroenteritis virus (TGEV), another porcine coronavirus, demonstrated that aerosolized TGEV was more stable at 30% RH vs. 90% RH (Kim et al., 2007). Importantly, many of these studies were conducted in aerosolized systems, which may not fully represent virus stability of solid surfaces such as filter paper. Nevertheless, the effect of RH on stability likewise applies to virus on surfaces. For instance, recovery of infectious TGEV from stainless steel showed a U-shaped RH effect, with greater stability at very low and very high RH relative to mid-range conditions. Specifically, at 4 °C, TGEV persisted for up to 28 days with little loss at 20% RH (< 1 x 10^0.5^ reduction), but a higher rate of inactivation was measured at 50% RH (1 × 10^3.5^ reduction) and 80% RH (1 × 10^3.2^ reduction) (Casanova et al., 2010).

A complicating factor when evaluating virus stability on environmental surfaces is the surface itself. For example, at ~25 °C and 40 - 55% RH, IAV half-lives were 10.3 hours on plain paper, 3.3 hours on inkjet paper, 3 hours on polystyrene, and 0.2 hours on plastic (Zhang et al., 2024). When characterized as “RNA abundance”, i.e., 2^-ΔCt^ × 1000, IAV RNA was more stable on polyethylene terephthalate, stainless steel, and glass after 24 hours at >80% RH versus <20% or 40 - 60% RH (Meng et al., 2022).

Thus, virus stability is a result of the interaction between temperature, RH, time, and the surface matrix. Study 1 examined the effect of temperature (5 °C, 20 °C, 35 °C) and RH (<20%, 40 - 65%, >75%) on the recovery of PRRSV, PEDV, and IAV RNA from 4 paper products over a 7-day observation period. The analysis showed that temperature was the dominant factor, with no significant effect attributed to RH, time, or paper product. Our findings were consistent with other previous reports demonstrating the strong influence of temperature on virus stability (**Table 4**). It should be noted, however, that many of these studies evaluated viral infectivity rather than RNA detection, which may not directly correspond to RT-qPCR based outcomes.

Based on the results of Study 1, Study 2 was designed to reduce experimental complexity by fixing temperature at 20 °C and focusing on a subset of paper types and viruses, thereby enabling a more detailed evaluation of RNA over an extended period of time, while retaining RH given its known influence on viral stability. Because IAV RNA was the most labile in Study 1, it was not included in Study 2. Thus, Study 2 expanded the observation period to 28 days and evaluated PRRSV and PEDV recovery from 2 paper products held at 20 °C and <20%, 40 - 65%, or >75% RH. Unexpectedly, among 228 swatches tested, 99.1% were positive for PRRSV or PEDV RNA across the 28-day period, albeit nonlinear exponential decay modeling revealed that decay rates increased with RH for both viruses. Notably, no evidence of decay was detected at <20% RH, indicating that low humidity stabilizes RNA on the surfaces evaluated in this study. This is consistent with previous reports in the refereed literature (Casanova et al., 2010; Zhang et al., 2024). The more pronounced RH-dependent effects observed in Study 2 compared to Study 1, including within the first 7 days, likely reflect both the extended observation period and improved methodological consistency. Study 1 exhibited greater variability in RNA recovery, which may have limited the detection of subtle RH effects over shorter timeframes. In contrast, modifications to the elution protocol in Study 2 improved consistency, which may have allowed RH-dependent differences in decay rates to be more clearly resolved. These findings are consistent with prior studies demonstrating that RH can influence virus stability, although such effects may only become apparent under conditions that minimize analytical variability and allow sufficient time for divergence in decay patterns.

Using the reported elution protocols for each paper product documented in prior work (Armenta-Leyva et al., 2025), paper product performance in terms of RNA recovery was broadly equivalent across the viruses evaluated. This lack of differentiation among paper types was consistent across analytical approaches. That is, no paper product provided higher or longer viral RNA detection in either Study 1 or Study 2. Given this outcome, cost becomes a consideration. The paper products evaluated in these studies ranged widely in price from ~$0.01 to ~$6.92 per swatch. This implies that careful attention to storage temperature and RH is more critical for reliable viral RNA detection than the choice of paper. Consistent virus detection across paper products in these controlled experiments suggests that filter paper merits further exploration as a practical, low-cost matrix for sample collection, storage, and laboratory testing, in settings that capture the complexity of field environments, including heterogeneous contamination, organic material, and airflow dynamics.

## Data Availability

The raw data supporting the conclusions of this article will be made available by the authors, without undue reservation.
